# An audit of licensed Zimbabwean radiology equipment resources as a measure of healthcare access and equity

**DOI:** 10.11604/pamj.2019.34.60.18935

**Published:** 2019-10-01

**Authors:** Tashinga Maboreke, Josephat Banhwa, Richard Denys Pitcher

**Affiliations:** 1 Division of Radiodiagnosis, Department of Medical Imaging and Clinical Oncology, Faculty of Medicine and Health Sciences, Stellenbosch University and Tygerberg Hospital, Cape Town, South Africa; 2 Radiation Protection Authority of Zimbabwe, 1 McCaw Drive, Avondale, Harare, Zimbabwe; 3 Department of Radiology, University of Zimbabwe, P.O. Box MP 167, Mount Pleasant, Harare, Zimbabwe

**Keywords:** Registered radiology equipment, universal health coverage

## Abstract

**Introduction:**

Approximately two-thirds of the world's population has no access to diagnostic imaging. Basic radiological services should be integral to universal health coverage. The World Health Organization postulates that one basic X-ray and ultrasound unit for every 50000 people will meet 90% of global imaging needs. However, there are limited country-level data on radiological resources, and little appreciation of how such data reflect access and equity within a healthcare system. The aim of this study was a detailed analysis of licensed Zimbabwean radiological equipment resources.

**Methods:**

The equipment database of the Radiation Protection Authority of Zimbabwe was interrogated. Resources were quantified as units/million people and compared by imaging modality, geographical region and healthcare sector. Zimbabwean resources were compared with published South African and Tanzanian data.

**Results:**

Public-sector access to X-ray units (11/10^6^ people) is approximately half the WHO recommendation (20/10^6^ people), and there exists a 5-fold disparity between the least- and best-resourced regions. Private-sector exceeds public-sector access by 16-fold. More than half Zimbabwe's radiology equipment (215/380 units, 57%) is in two cities, serving one-fifth of the population. Almost two-thirds of all units (243/380, 64%) are in the private sector, routinely accessible by approximately 10% of the population. Southern African country-level public-sector imaging resources broadly reflect national per capita healthcare expenditure.

**Conclusion:**

There exists an overall shortfall in basic radiological equipment resources in Zimbabwe, and inequitable distribution of existing resources. The national radiology equipment register can reflect access and equity in a healthcare system, while providing medium-term radiological planning data.

## Introduction

The United Nations 2030 Agenda for Sustainable Development, with 17 Sustainable Development Goals (SGDs), is a clarion call for unified global action to address our planet's stark inequalities. Health is addressed in the third SDG. Universal health coverage (the provision of quality, essential health services for all) is a key target [1,2]. Inequalities in global access to healthcare exist between and within nations. Between-country disparities are principally influenced by national wealth, which may be broadly stratified by World Bank income groupings. Low- and middle-income countries (LMICs) by World Bank criteria are home to more than 84% of the world's population, have 90% of the global burden of disease, but account for only 12% of global health spending [3]. The overwhelming majority of global deaths attributed to poverty and/or poor healthcare infrastructure occur in LMICs [4]. In-country health-care inequalities are largely attributable to disparities in resource distribution, with service provision to rural populations constituting a particular challenge [5,6]. Additionally, although private healthcare is playing an increasing role in service provision in all countries, differential access to private facilities contributes to in-country disparities, particularly in LMICs [7]. Healthcare technology, including diagnostic imaging, is acknowledged as an essential component of any healthcare system [5,8-10]. Basic radiological services, such as plain X-rays and ultrasound, are required for effective primary care [5,11-12]. Access to basic diagnostic imaging services should thus be seen as integral to achieving universal health coverage. The World Health Organization (WHO) recommends one X-ray and ultrasound unit for every 50000 people, or 20 units per million people, and postulates that this will meet ninety percent of global imaging needs [12,13]. This can serve as a yardstick to evaluate access to basic imaging services at country-level. Robust country-level data are thus required to assess the extent to which countries meet this target. However, there is a striking paucity of imaging resource data at country level. Although the WHO has published national estimates of high-end radiology equipment resources based on questionnaire surveys of member countries, these data do not include basic equipment such as general radiography and ultrasound units [14,15]. It is estimated that two-thirds of the world's population has no access to basic imaging services [16]. In May 2007, the 60^th^ UN World Health Assembly adopted Resolution 60.2^9^, urging member states to “collect, verify, update and exchange information on health technologies, in particular medical devices, as an aid to their prioritization of needs and allocation of resources”[17]. Notwithstanding this, there has been very little detailed work on in-country imaging resources, globally. The drivers and determinants of these resources remain poorly understood and the relationship between national healthcare expenditure, national health indicators and in-country access to diagnostic imaging has not been assessed. Additionally, there appears to be scant recognition of the potential role of registered diagnostic imaging equipment in reflecting healthcare access and equity within and between countries. Radiology equipment that emits ionizing radiation is generally licensed for use in a specific location that has been found to meet the infrastructural specifications for safe operation, such as adequate radiation shielding and appropriate electrical supply. Relocation of equipment typically requires re-licensing. Additionally, diagnostic imaging equipment may only be operated by registered radiation workers. An inventory of licensed equipment thus provides robust data on the number and distribution of units, as well as broader insights into the so-called “imaging enterprise” [16].

It is in this context that the Division of Radiodiagnosis in the Department of Medical Imaging and Clinical Oncology at Stellenbosch University embarked on an evaluation of the registered diagnostic radiology resources of Southern African countries. The current text focuses on Zimbabwe, and represents the third country-level study in the series. The first two studies reported data from South Africa (SA) and Tanzania, respectively [6,18]. Zimbabwe, is a land-locked, low-income country in sub-Saharan Africa. It has a predominantly rural population of approximately 13 million people, an area of 390757 square kilometres, and an overall population density of 33 people per square kilometre (Table 1). Administratively, the country has 8 provinces and 2 cities with provincial status (Harare and Bulawayo). The 2017 gross domestic product (GDP) was 17.8 billion US dollars (USD). Approximately 6% of GDP is spent on healthcare [19,20]. A primary care-based public healthcare sector predominates in Zimbabwe. Entry to the system is via approximately 1331 health centres, staffed by nursing sisters. Most communities are within 8 kilometres of such a facility. Secondary care is provided by approximately 64 urban/rural District Hospitals, which typically provide the first point of doctor-patient contact. These are largely government-run, but include some faith-based facilities. Eight Provincial Hospitals provide tertiary care, while quaternary care is at six Central Hospitals, most of which are teaching institutions. Approximately 10% of the Zimbabwean population has private medical insurance. There are urban private hospitals operated for profit, as well as rural hospitals run by large mining/farming companies for the benefit of their staff and their dependents. Since independence in 1980, private healthcare has grown at all levels. In particular, private practitioners have proliferated in the urban areas and in informal peri-urban settlements [19-24]. In the first 15 years after political independence, Zimbabwe developed one of the strongest economies and health systems in Southern Africa. However, from the mid-1990s public healthcare funding and infrastructure has declined, as a result of economic challenges, with steady erosion of previously achieved positive health indicators [23,24]. Zimbabwe has no formal, national policy on health technologies. Nonetheless, the Radiation Protection Authority of Zimbabwe (RPAZ) is the national statutory body responsible for registration of all healthcare equipment [25]. The aim of this study was a comprehensive analysis of licensed Zimbabwean diagnostic imaging equipment and comparison of Zimbabwean resources with the WHO guidelines on basic imaging services, and with recently published imaging data from other Southern African countries.

**Table 1:** Zimbabwean population by region

Province	population	area	population density
	(x 10^6^)	(m^2^)	(people/km^2^)
Harare	2.12	872	2435
Bulawayo	0.65	479	1364
Manicaland	1.75	36459	48
Masvingo	1.49	56566	26
Midlands	1.61	49166	33
Mashonaland West	1.50	57441	26
Mashonaland Central	1.15	28347	41
Mashonaland East	1.34	32230	42
Matabeleland North	0.75	75025	10
Matabeleland South	0.68	54172	13
Total	13.06	390757	33

## Methods

The study was conducted in Zimbabwe, in August 2016, in collaboration with the RPAZ. The official RPAZ database was systematically interrogated for all diagnostic imaging equipment in clinical use, including general radiography, fluoroscopy, computed tomography (CT), mammography, magnetic resonance (MR), angiography and positron emission tomography (PET)-CT units. Data were collated on a customized data sheet and stratified by imaging modality, geographical region, and health-care sector (public/private). Dental equipment was excluded. Non-profit, faith-based hospitals that serve all citizens, were classified as public sector facilities. Ten percent of the population were assumed to have private health insurance. For each modality, results were tabulated as equipment units per million people, for the country as a whole, by administrative region and by healthcare sector. Access to basic public-sector services was assessed by comparing Zimbabwean resources with the WHO guidelines for basic radiological equipment. Equity in the distribution of public-sector equipment was evaluated by comparing the least- and best-resourced administrative districts. The in-country disparity between public- and private-sector services was calculated for each modality. Zimbabwean resources were also compared with recently published country-level data from South Africa and Tanzania [7,13,14,22]. Aggregated data from recent World Bank and WHO resources were used for comparison of key healthcare economic and SDG indicators for Zimbabwe, South Africa and Tanzania [19,20,25-27].

## Results

**Registered Zimbabwean radiology equipment resources:** more than half of all Zimbabwe's radiology equipment units (215/380, 57%) are in the two major cities of Harare and Bulawayo, and almost two-thirds of all units (242/380, 64%) are in the private sector. Broadly, a cost-driven hierarchy of access to imaging is evident across public sector facilities, such that plain X-rays tend to be available from District Hospital level, CT at some Provincial/Central Hospitals and MR at selected Teaching Hospitals. However, there is no access to fluoroscopy and mammography outside the major cities. In four of the ten provinces, with a combined population of approximately 3.9 million people (30% of the total population), plain radiography is the only public sector imaging modality. The overall geographic distribution of private sector resources is similar to that of the public sector, with the proviso that in six of the ten provinces, plain radiography is the only available imaging modality (Table 2,Table 3).

**Table 2:** Registered Zimbabwean radiology equipment units by region, modality and healthcare sector

Modality		Harare	Bulawayo	Manicaland	Masvingo	Midlands	Mashonaland			Matabeleland		Total
							West	Central	East	North	South	
		units/10^6^ people										
x-ray	tot	68	51	11	20	16	10	17	10	19	15	26
	pub	7	29	8	14	8	11	14	9	11	15	11
	pvt	603	245	40	74	86	93	35	15	133	15	155
fluoro-scopy	tot	4.5	1.5	0	0	0	0	0	0	0	0	0.8
	pub	0.5	0	0	0	0	0	0	0	0	0	0.1
	pvt	38	15	0	0	0	0	0	0	0	0	7
mammo-graphy	tot	4	5	0	0	0	0	0	0	0	0	0.8
	pub	0.5	3	0	0	0	0	0	0	0	0	0.3
	pvt	33	15	0	0	0	0	0	0	0	0	6
CT	tot	5	5	1	0.7	1	0.7	0	0	0	0	1.5
	pub	0.5	3	0.6	0.7	0.7	0.8	0	0	0	0	0.6
	pvt	42	15	6	0	6	0	0	0	0	0	9
MR	tot	2	3	0	0	0	0	0	0	0	0	0.5
	pub	0.5	1.5	0	0	0	0	0	0	0	0	0.2
	pvt	14	15	0	0	0	0	0	0	0	0	3

**Table 3:** Registered Zimbabwean radiology equipment units per million people, by region, modality and healthcare sector

Modality		Harare	Bulawayo	Manicaland	Masvingo	Midlands	Mashonaland			Matabeland		Total
							West	Central	East	North	South	
		units (n)										
x-ray	Tot	142	33	19	30	25	29	19	13	14	10	334
	pub	14	17	12	19	11	15	15	11	8	9	131
	pvt	128	16	7	11	14	14	4	2	6	1	203
fluoro-scopy	Tot	9	1	0	0	0	0	0	0	0	0	10
	pub	1	0	0	0	0	0	0	0	0	0	1
	Pvt	8	1	0	0	0	0	0	0	0	0	9
mammo-graphy	Tot	8	3	0	0	0	0	0	0	0	0	11
	pub	1	2	0	0	0	0	0	0	0	0	3
	pvt	7	1	0	0	0	0	0	0	0	0	8
CT	Tot	10	3	2	1	2	1	0	0	0	0	19
	pub	1	2	1	1	1	1	0	0	0	0	7
	pvt	9	1	1	0	1	0	0	0	0	0	12
MR	Tot	4	2	0	0	0	0	0	0	0	0	6
	pub	1	1	0	0	0	0	0	0	0	0	2
	pvt	3	1	0	0	0	0	0	0	0	0	4

**Plain radiography:** although public sector equipment is available in all geographic regions, there is an approximately 5-fold discrepancy in access between the least-and best-resourced provinces. Only Bulawayo has the recommended WHO benchmark of at least 20 units per million people. Despite the combined public- and private-sector resources (25.7 units/10^6^ people) meeting the recommended WHO benchmark, there is a 16-fold discrepancy in access between the sectors. Public sector access (11 units/10^6^ people) is just over half the recommended WHO benchmark, while that in the private sector is almost 8-times the benchmark.

**Fluoroscopy:** there is very limited public sector access, with a single unit in Harare. More than 80% of Zimbabweans in the public health sector have no access to the modality. Although units are more readily available in the private sector these are all in the major cities. As a modality, fluoroscopy has the country's most striking disparity (69-fold) in access between the public and private sectors.

**Mammography:** units are confined to the major cities and 72% (8/11 units) are in the private sector. In eight provinces, covering 99% of the total land area, there is no access to mammography.

**Computed tomography:** almost seventy percent of equipment (13/19 units, 68%) is in the major cities, and more than 60% (12/19 units, 63%) in the private sector. The disparity in access between the public and private sectors (1:16) is comparable to that for plain radiography. The national ratio of plain X-ray to CT units (1:16-18) is similar in the public and private sectors.

**Magnetic resonance imaging:** the geographical distribution of units is similar to that of mammography, with two-thirds (4/6 units) in the private sector and a 15-fold discrepancy in access between the private and public sectors.

**Other modalities:** positron-emission tomography (PET)-CT and digital subtraction angiography (DSA) are unavailable.

**Comparison of radiological equipment resources for Tanzania, Zimbabwe and SA:** although Tanzania has the lowest quantum of national public sector resources, it has the most equitable distribution of basic equipment, and the lowest overall discrepancy in access between the public and private sectors (Table 4).

**Table 4:** Comparison of Zimbabwean, Tanzanian and South African registered radiology equipment resources, by modality and health sector

Modality		Tanzania	Zimbabwe	South Africa
		units/10^6^ people		
X-ray	total	9	26	35
	public	6	11	20
	private	26	160	104
	lowest: highest public sector regional density	1:2.2	1:5	1:2.6
	public: private	1:5	1:16	1:5
Fluoroscopy	total	1	0.8	6.6
	public	1	0.1	2.5
	private	2	7	27
	lowest: highest public sector regional density	1:2	0:0.5	1:9
	public: private	1:2	1:69	1:11
Mammography	total	0.3	0.8	5
	public	0.2	0.2	1.3
	private	0.6	6.1	22.3
	lowest: highest public sector regional density	0:0.5	0:1.7	0:2.6
	public: private	1:3	1:31	1:17
CT	total	0.42	1.5	5
	public	0.08	0.6	1.7
	private	2.	9	21
	lowest: highest public sector regional density	0:0.2	0:3.4	1:6.8
	public: private	1:27	1:16	1:12
MR	total	0.1	0.5	3
	public	0.05	0.2	0.3
	private	0.27	3.1	15
	lowest: highest public sector regional density	0:0.24	0:1.7	0:0.8
	public: private	1:5	1:15	1:46

**Comparison of demographic, economic and health indicator data for Tanzania, Zimbabwe, and SA:** national public sector imaging resources broadly reflect per capita healthcare expenditure, such that the lower the national expenditure, the lower the resources. However, the relationship is not linear. Additionally, despite having more imaging resources than Tanzania, Zimbabwe has inferior healthcare indicators (Table 5).

**Table 5:** Comparison of Zimbabwean, Tanzanian and South African demographic, economic and health indicator data

Parameter	Tanzania	Zimbabwe	South Africa
Total population (x 10^6^)	45	13	54
Rural population (%)	68	67	34
GDP (x 10^9^ USD)	52	18	350
GDP per capita (USD)	936	1079	6161
Health expenditure per capita (USD)	32	94	471
Total health expenditure as % GDP	6	10	8
Out-of-pocket health expenditure as % of total health expenditure	26	26	8
Population with private health insurance (%)	16	10	17
Maternal mortality/10^5^ live births	398	443	138
Neonatal mortality/10^3^ live births	19	24	11
Under 5 year mortality/10^3^ live births	49	71	41
Life expectancy	54	52	55
TB incidence/10^5^ people	306	242	834

## Discussion

This study provides useful medium-term planning data for the provision of basic radiological services, and the achievement of equity in Zimbabwe's public sector imaging resources. Based on the WHO guideline of 20 standard X-ray units per million people, our findings suggest an overall shortfall of approximately 9 units per million people in this sector. Additionally, the identification of a 5-fold discrepancy in concentration between the least- and best-resourced public-sector regions informs the optimal placement of any new equipment. Based on our analysis, the need is greatest in Matabeleland North, Harare, Manicaland and the Midlands. The WHO has estimated that approximately 90% of imaging requirements in resource-constrained environments can be provided by the basic modalities of general radiography and ultrasound [12,13]. Conversely, it is anticipated that more sophisticated investigations such as fluoroscopy, mammography, CT, and MR will constitute approximately 10% of investigations in such settings. The optimal concentration of these modalities has not been defined for any healthcare setting. However, extrapolation of the WHO guidelines suggests that 1-2 units/million people are required for modalities of intermediate cost, such as fluoroscopy, mammography and CT. Our findings show that the provision of one fluoroscopy, mammography and CT unit in all Provincial Hospitals would substantially enhance Zimbabwe's national radiological capacity. This would constitute a realistic and achievable medium-term goal, for which accurate cost projections are possible. Additionally, the national deficit in radiological equipment can serve as a proxy estimate of the additional human resources, by way of radiographers, radiologists, and medical physicists required to coordinate a more equitable public-sector radiological service. A national registry of radiology equipment reflects additional aspects of the healthcare system. The Zimbabwean registry demonstrates very high concentrations of private-sector plain radiographic units in the main cities. Concentrations in Harare and Bulawayo are 30- and 12-times the WHO guideline respectively, while there is an 80-fold disparity between public-and private sector resources in Harare. The plethora of basic imaging equipment in the urban private sector is likely a consequence of the dual impact of a steady decline in public sector infrastructure and rapid urbanization over the past two decades. Between 1982 and 2012 the population of Harare more than doubled, resulting in an influx of private medical practitioners, many of whom invested in their own basic imaging equipment [19,28]. While this is testimony to the pivotal role of basic imaging in primary care, it also reflects a potential regulatory vacuum in this domain. Similar trends are evident in private-sector CT access in Harare, where the unit concentration (51/10^6^ people) is almost four-times the Organization for Economic Co-operation and Development (OECD) average (13.3/10^6^), more than double that in the SA private sector (20/10^6^) and 25% higher than that in the United States (40/10^6^ people) [7]. Our findings suggest that appropriate legislative and regulatory measures are required to rationalize Zimbabwean radiology equipment in the urban private sector. While current unit concentrations for plain radiography and CT may promote access to imaging, the apparent oversupply potentiates self-referral, over-utilization, unnecessary exposure to ionizing radiation and burgeoning healthcare costs [29-33]. Of note, Zimbabwe is one of 90 WHO member states (90/174, 52%) that have no national health technology policy [17].

Our finding that SA, a high middle-income country by World Bank criteria, has a greater overall density of diagnostic imaging equipment than Zimbabwe and Tanzania, both low-income countries, is intuitive. The same is true for our demonstration that public sector imaging resources broadly reflect national per capita healthcare expenditure. However, the relationship between healthcare expenditure, diagnostic imaging equipment resources and healthcare indicators is more complex. Despite Zimbabwe's annual per capita healthcare expenditure, and its public-sector plain X-ray equipment resources being three-times that of Tanzania, all major Zimbabwean healthcare indicators are inferior to those of Tanzania. Similarly, despite SA's annual per capita healthcare expenditure exceeding that of Tanzania by 16-fold and the average density of SA public sector imaging resources surpassing those of Tanzania by 30-fold across the modalities, SA healthcare indicators are not commensurate. While these observations underscore the “complex, manifold and intricate” nature of health systems [17], it is salutary to note the equitable distribution of basic Tanzanian public-sector X-ray equipment. It is possible that the distribution of basic public sector radiological equipment in Tanzania is a reflection of equitable distribution of other key resources in the healthcare system. Of note, the geographic mal-distribution of Zimbabwean healthcare resources has been implicated in the failure of the national public-sector referral system. Patients have been observed to bypass their nearest peripheral facility due to lack, or perceived lack, of basic resources, in preference for care at a higher-level public facility or in the private sector [17,34]. The strength of this quantitative work is its foundation on the RPAZ official database of registered diagnostic imaging equipment, together with the RPAZ's full collaboration in the project. A limitation is the absence of a qualitative component to assess equipment functionality. It is possible that this introduced an overall positive bias in Zimbabwe's public sector resources. A further limitation is the absence of ultrasound data. Ultrasound equipment is not registered, since it does not involve ionizing radiation. This limitation is common to all current analyses of national diagnostic imaging resources and is a major constraint in the evaluation of the imaging capacity in LMICs, where ultrasound plays a potentially pivotal role. This paper represents the third in a series of planned manuscripts analyzing registered radiological equipment resources in individual Southern African countries. It is hoped that the paper provides some insight into how such analyses contribute to the discourse on access and equity in healthcare. Such work can potentially be integrated into future, broader, health service and health systems analyses, and is becoming increasingly important as diagnostic imaging assumes an ever more pivotal position at all levels of health care delivery. Furthermore, it is hoped that this document will stimulate similar analyses in other WHO regions and provide a framework for such work, thereby enhancing understanding of the determinants of imaging resources and utilization at country-level.

## Conclusion

There exists an overall shortfall in basic radiological equipment resources in Zimbabwe, and there is inequitable distribution of existing resources. This study highlights the role of a national audit of registered radiology equipment in defining country-level health coverage and equity, and providing medium-term planning data.

### What is known about this topic

- Basic radiological services, such as plain X-rays and ultrasound, are required for effective primary care;
- The World Health Organization (WHO) postulates that one basic X-ray and ultrasound unit for every 50,000 people will meet 90% of global imaging needs;
- There is a paucity of detailed data on in-country registered radiological equipment resources, globally.

### What this study adds

- It shows that access to basic X-ray equipment (11 units/10 ^6^people) in Zimbabwe's public healthcare sector is approximately half the recommended WHO benchmark (20 units/10^6^ people);
- It highlights the 5-fold disparity in basic public-sector X-ray resources between the least- and best-resourced regions, and the 16-fold disparity between public- and private-sector access to basic X-ray services;
- It illustrates how a national audit of registered radiology equipment can provide insights into country-level health coverage and equity, and assist in medium-term healthcare planning, to meet WHO radiological benchmarks.

## Competing interests

The authors declare no competing interests.
